# On the Coupling Between Cosmological Dynamics and Quantum Behavior: A Multiscale Thermodynamic Framework

**DOI:** 10.3390/e27090976

**Published:** 2025-09-18

**Authors:** Andreas Warkentin

**Affiliations:** Institute of Mechanics, University of Kassel, 34125 Kassel, Germany; warkentin@uni-kassel.de or physics@drwaan.com

**Keywords:** thermodynamics, multiscale, quantum mechanics, quantum physics, multiscale coupling, non-equilibrium thermodynamics

## Abstract

A multiscale thermodynamic model is considered, in which cosmological dynamics enforce persistent non-equilibrium conditions through recursive energy exchange across hierarchically ordered subsystems. The internal energy of each subsystem is recursively determined by energetic interactions with its subcomponents, forming a nested hierarchy extending up to cosmological scales. The total energy of the universe is assumed to be constant, imposing global consistency conditions on local dynamics. On the quantum scale, subsystems remain thermodynamically constrained in their accessible state space due to the unresolved energetic embedding imposed by higher-order couplings. As a result, quantum behavior is interpreted as an effective projection of unresolved thermodynamic interactions. In this view, the wave function serves as a mathematical representation of a subsystem’s thermodynamic embedding, summarizing the unresolved energetic couplings with its environment, as shaped by recursive interactions across cosmological and microscopic scales. Phenomena such as zero-point energy and vacuum fluctuations are thereby understood as residual effects of structural energy constraints. Classical mechanics arises as a limiting case under full energetic resolution, while the quantum formalism reflects thermodynamic incompleteness. This formulation bridges statistical mechanics and quantum theory without metaphysical assumptions. It remains fully compatible with standard formalism, offering a thermodynamic interpretation based solely on energy conservation and hierarchical organization. All effects arise from scale-dependent resolution, not from violations of established physics.

## 1. Introduction

Unifying gravity with quantum physics remains one of the central challenges in theoretical physics. While general relativity (GR) passes all tests at macroscopic scales, its merger with quantum theory remains elusive. String theory, though extensively developed, fails to yield a unique low-energy limit, limiting its predictive power [1,2,3]. Loop quantum gravity (LQG) offers a background-independent quantisation of gravity and predicts discrete spectra for geometric operators [4,5]. However, it struggles to incorporate standard model fields and to recover classical GR in the semiclassical limit. An alternative class of models modifies gravity or introduces new dynamical fields. Scalar–tensor theories, including quintessence, invoke a dynamical scalar field to explain late-time cosmic acceleration [6,7], offering an alternative to a cosmological constant and addressing fine-tuning and coincidence problems [8]. Many modified gravity models, such as f(R) and Horndeski theories, can be recasted as scalar–tensor frameworks with non-minimal couplings [9]. Within quantum gravity, two complementary programs stand out. The asymptotic-safety scenario posits a non-perturbative UV fixed point that makes gravity fundamentally renormalizable [10,11]. Renormalization-group flows drive gravitational couplings to finite values at Planckian scales, while IR fixed points can trigger spontaneous scale symmetry breaking, generating particle masses and dynamically selecting fundamental scales [12,13]. A concrete realization is the cosmon framework, where a time-dependent scalar field remains scale-invariant at both fixed points but breaks symmetry between the fixed points, which accounts for inflation and dark energy without fine-tuning or extra fields [14,15]. Second, approaches reinterpret gravity not as a fundamental force but as a macroscopic consequence of underlying thermodynamic or informational structure. Entropic and holographic scenarios derive Newtonian and Einsteinian dynamics from thermodynamic or information-theoretic properties of microscopic degrees of freedom, linking quantum information to geometry [16,17,18,19]. Recently, a consistent framework coupling classical gravity to quantum matter was proposed, avoiding the need to quantize the metric [20]. The theory is linear, completely positive, and trace-preserving, reproducing Einstein’s equations in the classical limit. Crucially, fundamental stochasticity in both geometry and matter allows the model to evade no-go theorems while naturally inducing decoherence without invoking measurement postulates. In a related framework, ’t Hooft models systems as discrete states evolving under a unitary matrix, where quantum mechanics appears as an effective description of underlying deterministic dynamics [21].

A multiscale framework by Wetterich links particle mass generation and cosmic evolution to a time-varying scalar field, thereby avoiding an initial singularity [12]. Similarly, Verlinde’s entropic approach reframes gravity as an information-based ordering principle rather than a fundamental force [16]. While these frameworks offer theoretical insights, none has yet received decisive experimental support.

Rather than relying on a specific microscopic origin such as entropic, holographic, or informational mechanisms [12,16,17,21], the present model offers a structural unification of these perspectives through a dynamic, energy-based interpretation in which collective gravitational behavior arises organically from recursive multiscale organization. Crucially, the local internal energy at each scale is defined as a functional of the internal energies of its subsystems, establishing a fully nested energy hierarchy. This recursive structure enables natural coupling across all physical scales and provides a mechanism for quantum behavior arising from structurally constrained multiscale interactions governed by cosmological dynamics. The multiscale model thus invites a reconsideration of foundational assumptions from a dynamic, network-based viewpoint.

The importance of multiscale modeling has long been recognized in other fields of science, notably in chemistry, where the 2013 Nobel Prize was awarded to Martin Karplus, Michael Levitt, and Arieh Warshel for pioneering methods that couple quantum and classical regimes to simulate complex systems efficiently [22,23,24,25]. Building on this principle, the present framework extends the concept of cross-scale coupling beyond molecular systems by establishing recursive multiscale interaction as a structural foundation of physical organization. This formulation introduces a dynamical paradigm in which Newtonian mechanics arises as a limiting case of recursively structured multiscale couplings, rather than being postulated a priori. Gravitational theory remains operationally valid at macroscopic scales, yet its source terms are recast as effective expressions of deeply nested energy architectures across physical hierarchies.

Modeling decisions, such as which scales to resolve and with what precision, depend on the application. Quantum mechanical, mesoscopic, and classical descriptions may coexist across spatial regions without conceptual conflict, as long as coupling remains consistent. From this broader perspective, chemical multiscale modeling can be viewed as a mature, domain-specific realization of a more general paradigm, one that integrates macroscopic behavior with microscopic structure through coherent scale-bridging strategies. Building on this idea, we now construct the model from a thermodynamic viewpoint, beginning with a classical analogy and postponing field-theoretic or continuum assumptions.

## 2. The Recursive Thermodynamic Model

Before introducing the mathematical formulation, we clarify the terminology and conceptual framing used throughout this work. Terms such as “nested structure” and “hierarchy” are employed in a technical sense, grounded in statistical mechanics and multiscale systems theory. By nested structure, we refer to a recursive organization in which each subsystem is thermodynamically embedded within, and constrained by, a superordinate system. This top-down influence defines the accessible state space of each level. The term hierarchy, in this context, denotes a functional ordering of energetically interacting systems across multiple scales, where each level contributes to the constraint structure of the next.

The proposed approach is based on two simple postulates:

**Assumption** **1.** *The universe is regarded as an isolated thermodynamic system $S_{0}$ with no other systems of the same hierarchical level. Consequently, no potential energy contributions arising from interactions with other systems are possible, and, consistent with classical energy conservation, no collective motion (i.e., kinetic energy of the system) can arise. Its total energy, therefore, equals the internal energy $U_{0}$, which is not further specified at this point, without loss of generality. Accordingly, the rate of internal energy satisfies*

(1) $$\frac{d}{dt}U_{0}\;=:\;{\dot{U}}_{0}=0\;.$$

*This is interpreted as the minimal requirement for the universe.*

Although this formulation may appear trivial at first sight, the system at the highest hierarchical level has no external environment; hence, no heat flux, no external work, and no potential energy can be defined. This is not a limitation but the minimal thermodynamic requirement for an isolated universe.

To prepare the recursive formulation of internal energy, we first provide a definition of a system as used in this work.

**Definition** **1** (System)**.** *Throughout this work, a system denotes an energetically defined unit at a given hierarchical level. Each system can act simultaneously as a composite of entities on lower hierarchical levels and as a constituent of higher-level structures.*

The specific examples mentioned later, such as astronomical systems, macroscopic matter, or composite particles, are not to be read as alternative concepts but as illustrative realizations of the general definition of a system introduced above.

Since the universe as constructed in Assumption 1 is initially empty, we define its structure through its internal energy, composed of the total energy contributions of its subsystems. The internal energy can more formally be expressed as follows:

**Assumption** **2.** *Extending the concept of internal energy from Assumption 1, all astronomical systems, macroscopic matter, and composite particles, except elementary particles, which are regarded as structureless, possess an internal energy U, which is defined as*

(2) $$U=\sum_{\alpha }E_{\alpha }=\sum_{\alpha }U_{\alpha }^{\mathrm{loc}}+H_{\alpha }\;,$$

*where $U_{\alpha }^{\mathrm{loc}}$ is the local internal energy of subsystem α, and $H_{\alpha }=K_{\alpha }+P_{\alpha }$ is the Hamilton function, with $K_{\alpha }$ and $P_{\alpha }$ denoting, respectively, the kinetic and potential contributions of the corresponding subsystems. Furthermore, each $U_{\alpha }^{\mathrm{loc}}$ is defined in the same manner as in* (2)*, acting as a functional of the family of all recursively nested energy contributions of its subsystems.*

*Importantly, the potential energy terms $P_{\alpha }$ must be understood as referring to the interactions between the systems included within the subsystem α.*

In this formulation, the energies are treated as globally integrated quantities but may be understood as arising from spatial integration over corresponding field variables. A local, differential representation of the first law, as is common in continuum thermodynamics, is not used here. When point-like particles are considered, the continuum assumption may be partially dropped, and expressions known from classical mechanics are used in place of volumetric integrals.

**Remark** **1** (regarding Assumption 2)**.** *More formally, U is defined as a recursively nested family of energy contributions ${\left\{E_{\alpha }\right\}}_{\alpha }$ where each element $E_{\alpha }$ depends on a local internal energy $U_{\alpha }^{\mathrm{loc}}$, itself determined by a subordinate family ${\left\{E_{\alpha \beta }\right\}}_{\beta }$ at the next sublevel*

(3) $$\begin{array}{c} {\left\{E_{\alpha }(U_{\alpha }^{\mathrm{loc}}({\left\{E_{\alpha \beta }\right\}}_{\beta }))\right\}}_{\alpha }\;. \end{array}$$

*Thereby, a fully nested nonlinear hierarchical structure is established, where α∈{1,…,A}⊂N,A∈N, denotes the contributions at the first hierarchical sublevel (comprising A subsystems), and αβ, with β∈N, refers to the contributions at the sublevel of subsystem α. This can be quantitatively expressed for the first two scales as follows:*

(4) $$\begin{array}{c} U=\sum_{\alpha =1}^{A}E_{\alpha }(U_{\alpha }^{\mathrm{loc}}\left({\left\{E_{\alpha \beta }\right\}}_{\beta }\right)),\;\mathrm{where}\;E_{\alpha }=U_{\alpha }^{\mathrm{loc}}\left({\left\{E_{\alpha \beta }\right\}}_{\beta }\right)+H_{\alpha }\;. \end{array}$$

*This procedure can be straightforwardly generalized to more than two hierarchical scales by considering*

(5) $$\begin{array}{c} {\left\{E_{\alpha }(U_{\alpha }^{\mathrm{loc}}({\left\{E_{\alpha \beta }(U_{\alpha \beta }^{\mathrm{loc}}({\left\{E_{\alpha \beta \gamma }\left(\ldots \right)\right\}}_{\gamma }))\right\}}_{\beta }))\right\}}_{\alpha }. \end{array}$$

*In general, each local internal energy $U_{\alpha \beta \cdots \xi }$ is defined analogously in terms of its own subsystems,*

$$U_{\alpha \beta \cdots \xi }=\sum_{\zeta =1}^{Z(\xi )}E_{\alpha \beta \cdots \xi \zeta },$$

*thereby enabling unrestricted hierarchical recursion and capturing the multiscale nesting of energy contributions. Obviously, the recursive nesting terminates trivially at elementary particles, which are regarded as structureless entities without internal degrees of freedom.*

The second postulate serves as the foundational hypothesis of this framework, implying that each energy contribution on one level functions as a structurally constrained basis for further decompositions. This establishes nonlinear, recursively coupled interactions across scales, from cosmic to quantum domains. The recursive thermodynamic framework enforces strict global energy conservation across all scales. This necessarily implies a deterministic structure, as no stochastic degrees of freedom remain outside the hierarchy. While such determinism could be labeled ‘superdeterminism’, we stress that it is not introduced as an additional hypothesis but follows directly from energy conservation.

### 2.1. Internal Energy of the Universe

Based on the two postulates, the internal energy of the universe $U_{0}$ is defined across multiple hierarchical scales as

(6) $$\begin{array}{c} \begin{array}{cc} U_{0}\;\overset{!}{=}\;\mathrm{const}.\; & =\sum_{\alpha =1}^{J_{0}}E_{\alpha }\\ \; & =\sum_{\alpha =1}^{J}H_{\alpha }+U_{\alpha }^{\mathrm{loc}}\\ \; & =\sum_{\alpha =1}^{J_{0}}\left(H_{\alpha }+\sum_{\beta =1}^{J(\alpha )}(H_{\alpha \beta }+\sum_{\gamma =1}^{J(\beta )}(H_{\alpha \beta \gamma }+\ldots +U_{\alpha \beta \gamma \ldots \zeta }^{\mathrm{loc}}))\right)\;, \end{array} \end{array}$$

abbreviated as

(7) $$\begin{array}{c} \sum_{\alpha ,\beta ,\gamma ,\ldots ,\zeta }^{J(0,\alpha ,\beta ,\gamma ,\ldots ,\zeta )}\left(H_{\alpha \beta \gamma \ldots \zeta }+U_{\alpha \beta \gamma \ldots \zeta }^{\mathrm{loc}}\right)=:A_{s}\;, \end{array}$$

where A is introduced as an energy operator, and the index *s* encodes the hierarchical depth (i.e., the number of scales from the first level that are taken into account). For the universe $S_{0}$, taking all *S* scales into account, there is a further definition

(8) $$\begin{array}{c} A_{S}=U_{0}\overset{!}{=}\mathrm{const}.\;. \end{array}$$

### 2.2. Further Consequences Resulting from the Recursive Model

Each subsystem obeys a local form of the first law

(9) $$\begin{array}{c} \dot{E}=\dot{U}+\dot{H}=\dot{W}+\dot{Q}\;, \end{array}$$

yet remains embedded in a globally coupled thermodynamic structure, as given in Equation (8). Consequently, no subsystem evolves independently: cosmic-scale variations, such as orbital motion, solar output, or gravitational shifts, propagate downwards and modulate internal energy states across scales.

This has direct thermodynamic implications. According to the Clausius–Duhem inequality of rational thermodynamics, [26]

(10) $$\begin{array}{c} T{\dot{s}}^{\mathrm{irr}}=T\dot{s}-P+\dot{u}+\frac{1}{T}\vec{q}\cdot \vec{\nabla }T\geq 0\;, \end{array}$$

the irreversible part of the entropy rate is directly proportional to the rate of internal energy accumulation. Here, ${\dot{s}}^{\mathrm{irr}}$ denotes the irreversible part of the entropy rate, $\dot{s}$ denotes the total entropy rate, P denotes the power density, $\dot{u}$ denotes the rate of internal energy accumulation, $\vec{q}$ denotes the heat flux vector, and $\vec{\nabla }T$ denotes the temperature gradient. All quantities are defined per unit volume, as is common in continuum formulation of balance equations, e.g., $U=\int_{\Omega }udV$. Hence, a total equilibrium state is thermodynamically inaccessible when internal energy is hierarchically coupled across scales and conserved at the highest level, as required by Assumptions 1 and 2. This inaccessibility is not due to external disturbances but follows structurally: recursive coupling prevents the closure of entropy production at any hierarchical level. Each subsystem, therefore, persists in a state of compensated disequilibrium enforced by higher-level embedding, while interactions between systems of the same level are mediated through their common higher-level structure, a condition that distinguishes the present framework from conventional treatments, where non-equilibrium is regarded as contingent rather than structural.

In local formulations, global couplings remain implicitly embedded, much like boundary conditions in classical mechanics encode global constraints into local motion, or how path integrals embed the full history of all paths into the current amplitude. Effective parameters such as mass, energy, or charge must then absorb multiscale influences by construction. Effective physical parameters such as mass and inertia (or electric charge and energy) must then, by construction, absorb and reflect multiscale influences from these nested structures. In particular, experimentally accessible ratios such as the mass-to-charge ratio m/q may serve as sensitive probes for underlying hierarchical effects. The resulting structure acts as an informational mediator across scales, much like spacetime in general relativity encodes and transmits the mutual dependence between energy distributions and geometric curvature.

## 3. Derivation of Newtonian Laws from Recursive Energy Structures

In this framework, Newton’s axioms are obtained as structurally constrained limiting cases. The first law follows by considering a universe containing only a single system with ${\dot{U}}_{0}={\dot{E}}_{1}=0$ (point mass). From this, the classical formulation of inertia naturally arises

(11) $$\begin{array}{c} {\dot{K}}_{1}=\dot{\vec{p}}\cdot \dot{\vec{x}}=0\;\Rightarrow \;\dot{\vec{p}}=0\;\forall \dot{x}\neq 0\;, \end{array}$$

where $\dot{\vec{x}}$ and $\dot{\vec{p}}$ are referring to the vectors of velocity and linear momentum. Without the minimum requirement for the universe, the result is not tenable without detailed statements about the work performed on the system and the transferred heat. If we further consider the case of two point-mass systems in the universe from the perspective of classical physics, the following well-known relationships emerge:

(12) $$\begin{array}{c} {\vec{F}}_{1}=-{\vec{F}}_{2}\;\mathrm{and}\;m_{i}{\ddot{\vec{x}}}_{i}={\vec{F}}_{i}^{G}\;\forall \;{\dot{x}}_{i}\;\;\mathrm{with}\;i=1,2\;. \end{array}$$

These relations follow entirely without invoking Newton’s axioms or assuming energy conservation. Instead, they result directly from the minimum condition for the universe (Assumption 1), together with Assumption 2, via

(13) $$\begin{array}{c} \frac{d}{dt}A_{S}=\sum_{\alpha =1}^{2}{\dot{K}}_{\alpha }+{\dot{P}}_{\alpha }={\dot{\vec{x}}}_{1}\left(m_{1}{\ddot{\vec{x}}}_{1}-G\frac{m_{1}m_{2}}{r^{2}}\right)+{\dot{\vec{x}}}_{2}\left(m_{2}{\ddot{\vec{x}}}_{2}+G\frac{m_{1}m_{2}}{r^{2}}\right)\overset{!}{=}0\;. \end{array}$$

This holds for all velocities. Here, $r=\left|\right|{\vec{x}}_{1}-{\vec{x}}_{2}\left|\right|$ and $F^{G}$ is the Newtonian gravitational force. If the above is interpreted as a derivation by prefixing Assumptions 1 and 2, then the foundational role typically ascribed to these equations becomes questionable. This demonstrates that Newton’s second and third laws arise as consistency conditions of recursive multiscale couplings, rather than as independent postulates. This equation no longer applies if the system is not a point mass and, therefore, has a non-zero internal energy.

## 4. Hierarchical Energy Coupling and Local Observables

### 4.1. Thermodynamic Fluctuations Across Scales

Illustrating structural interdependence across scales, we consider a molecule on Earth $E_{E1}^{M}$ as a subsystem in the multiscale structure of the planet. The first law of thermodynamics, in conjunction with Earth’s internal energy, according to Assumption 2, provides

(14) $$\begin{array}{c} {\dot{E}}_{E1}^{M}={\dot{W}}_{E1}^{M}+{\dot{Q}}_{E1}^{M}={\dot{U}}^{E}-\left(\sum_{\beta =2}^{J_{E}}{\dot{U}}_{E\beta }+{\dot{H}}_{E\beta }\right)=:{\dot{U}}^{E}-{\dot{U}}_{E}^{\mathrm{residual}}\;. \end{array}$$

The indices M and E indicate the contributions of the molecule and the Earth. Obviously, the total energy of the molecule $E_{E1}^{M}$ changes in the same way as the difference $U_{E}-U_{E}^{\mathrm{residual}}$, e.g., when the Earth adopts a different orbit and thereby alters its energy level, or when $U_{E}$ and $U_{E}^{\mathrm{residual}}$ become identical or both vanish. Due to strong couplings, $U_{E}$ and $U_{E}^{\mathrm{residual}}$ cannot be treated as independent. Temporarily relaxing this constraint allows hypothetical scenarios involving changes in the velocity of the considered material subsystem. Large masses and high velocities significantly affect this balance, forcing the system to reduce its energy and, in extreme cases, perturbing the orbital dynamics of the carrier system. Such recursive feedback propagates across structurally coupled systems, both laterally and vertically, triggering scale-spanning energy reconfigurations throughout the nested hierarchy. This conceptual exploration underscores that local energy fluctuations cannot remain isolated within the total system and illustrates why the universe, given its vast energy content, large masses, and high velocities on average, exhibits remarkable large-scale stability. The macroscopic influence on the microscale becomes particularly visible when ${\dot{U}}_{E}$ is substituted in Equation (14) via the local first law, applied to the Earth system

(15) $$\begin{array}{c} {\dot{E}}_{E1}^{M}={\dot{W}}_{E1}^{M}+{\dot{Q}}_{E1}^{M}={\dot{W}}_{E}+{\dot{Q}}_{E}-{\dot{H}}_{E}-{\dot{U}}_{E}^{\mathrm{residual}}\;. \end{array}$$

It becomes obvious that the external energies of the Earth, as well as the work performed on the Earth and the heat flux across Earth’s surface, have an influence on the behavior of all matter and its subsystems.

#### Brownian Dynamics Under the Influence of Cosmological Fluctuations

Moreover, Equations (14) and (15) suggest that classical Brownian motion may not be purely stochastic in origin, but rather a local thermodynamic response to unresolved energy redistribution across scales. This becomes evident when recast in the form of a Langevin-type equation as follows:

(16) $$\begin{array}{c} m\ddot{x}=\frac{1}{\dot{x}}\left({\dot{W}}_{E}+{\dot{Q}}_{E}-{\dot{H}}_{E}-{\dot{U}}_{E}^{\mathrm{residual}}-{\dot{P}}_{E1}^{M}-{\dot{U}}_{E1}^{M}\right)\;. \end{array}$$

Whereas conventional models like the Langevin approach treat friction and noise as externally imposed stochastic inputs, this framework derives both as intrinsic manifestations of recursive multiscale energy exchange. This perspective generalizes the fluctuation–dissipation relation and reveals how nested thermodynamic structures systematically constrain the accessible dynamics of embedded systems.

### 4.2. Cosmic Effects on Quantum Systems

For a refined understanding of quantum behavior, we start by examining Earth’s internal energy, which, when resolved to quantum scales following Assumption 2, reveals the following structure:

(17) $$\begin{array}{c} \begin{array}{c} U^{E}=\sum_{\alpha =1}^{J}(H_{\alpha }^{E}+\sum_{\beta =1}^{J(\alpha )}(H_{\alpha \beta }^{E}+\sum_{\gamma =1}^{J(\beta )}(H_{\alpha \beta \gamma }^{E}+\sum_{\gamma =1}^{J(\gamma )}(H_{\alpha \beta \gamma \delta }^{E}+U_{\alpha \beta \gamma \delta }^{E,\mathrm{loc}}))))\;. \end{array} \end{array}$$

The electron of interest, e.g., in quantum level 1111, is removed from its interacting background, and its energy is expressed in isolated form as

(18) $$\begin{array}{c} U_{1111}^{E}+H_{1111}^{E}=E_{1111}^{E}\;. \end{array}$$

This representation is necessarily incomplete, since, in practice, the electron cannot be fully decoupled from multiscale interactions with its environment. Moreover, due to the principled inaccessibility of fully resolving all multiscale interactions in Equation (17), the unresolved contributions from these recursive couplings must be incorporated effectively.

By necessity rather than choice, the electron on this scale is represented by a wave function; it is the minimal description that (i) reproduces bound-state spectra, interference, and tunneling, (ii) ensures probability conservation and unitary time evolution generated by H, and (iii) reduces to Hamilton–Jacobi dynamics once all unresolved couplings are resolved. In this framework, the wave function provides a mathematically tractable encoding of unresolved multiscale influences, such that quantum behavior appears as a functional consequence of thermodynamic constraints imposed by the recursively coupled hierarchy.

Neglecting spin and exchange terms for simplicity, the effective dynamics then take the standard form

(19) $$\begin{array}{c} H_{1111}^{E}\;\phi =E_{1111}^{E}\;\phi \;, \end{array}$$

where *H* becomes an operator H. The kinetic term retains its conventional structure and is unaffected by the interpretational shift introduced here. From this point of view, the Schrödinger equation describes an effective subsystem governed by constraints arising from unresolved multiscale couplings beyond its descriptive scale. This connects to [21], where quantum mechanics is treated as an effective description of underlying deterministic dynamics, while the present framework specifies this description in terms of unresolved energetic structure within a multiscale hierarchy.

#### Quantitative Implications

As a direct ceiling, cosmological modulation of bound quantum systems can be bounded with the Hubble rate,

$$\left|\frac{\dot{\nu }}{\nu }\right|≲H_{0}∼10^{-10}\;{\mathrm{yr}}^{-1},$$

with recursive attenuation suppressing the effect by over 30 orders of magnitude at atomic scales; see Appendix B.1. For typical electronic transitions, this yields

$$\left|\frac{\dot{\nu }}{\nu }\right|≲10^{-43}\;{\mathrm{yr}}^{-1},$$

which is fully consistent with current atomic clock bounds (<$10^{-18}\;{\mathrm{yr}}^{-1}$). Details of the derivation are given in Appendix B.1.

### 4.3. Zero-Point Energy from Unresolved Recursive Thermodynamic Embedding

The Hamilton function of an ideal/monoatomic gas is given by the following equation:

(20) $$\begin{array}{c} H_{\alpha }=\frac{3}{2}Nk_{B}T+P\;. \end{array}$$

Here, *N* is the particle number of the considered system, and $k_{B}$ is the Boltzmann constant. If the temperature is set to zero, the Hamilton function vanishes, but the total energy does not disappear as long as the substructures β possess quantum mechanical degrees of freedom $\frac{1}{2}\hbar \omega_{\alpha \beta }>0$ per mode

(21) $$\begin{array}{c} \begin{array}{cc} E_{\alpha }\left(T=0\right)=U_{\alpha }+0+P_{\alpha }\; & =\sum_{\beta }U_{\alpha \beta }+H_{\alpha \beta }+P_{\alpha }\\ \; & =\sum_{\beta }\frac{1}{2}\hbar \omega_{\alpha \beta }+P_{\alpha \beta }+P_{\alpha }\geq 0\;. \end{array} \end{array}$$

This equation is embedded within the multiscale model and must also satisfy the equations governing the higher-order systems even if $P_{\alpha }$ and $P_{\alpha \beta }$ are zero; thus, it is evident that it cannot vanish; it could only do so if there were no further cosmic energy changes, implying an absolutely static universe. Consequently, within this recursive multiscale framework, a non-thermal energy remains that is not attributable to statistical thermodynamics, but it reflects unresolved energetic dependencies across hierarchically coupled systems. While this residual energy structurally resembles quantum mechanical zero-point energy, it originates from deterministic nesting within the multiscale structure.

### 4.4. Remarks on the Uncertainty Principle of Quantum Physics

In this framework, quantum uncertainty does not reflect intrinsic randomness; it arises from scale-dependent limitations in resolving energetic couplings within a recursively nested thermodynamic structure. The Heisenberg principle reflects these structural constraints: any localization perturbs the energetic embedding of a system, forcing a redistribution of unresolved degrees of freedom.

This applies even to structureless particles such as electrons, which—despite nominal isolation—remain thermodynamically coupled to their environment. Attempts to resolve their intrinsic dynamics inevitably inject measurement energy that exceeds the amplitude of cosmological background modulations, thereby altering the system itself.

Zero-point energy is interpreted analogously—it reflects residual, non-thermal contributions from unresolved substructures within the energy hierarchy, as shown in Equation (21). Even at zero temperature, this energy remains as a thermodynamic consequence of incomplete energetic resolution. Uncertainty and vacuum energy thus represent functional consequences of unresolved multiscale coupling—grounded in thermodynamic structure, not in quantum postulates.

## 5. Constraining the Energy–Momentum Tensor Through Recursive Thermodynamic Structure

Having established the recursive thermodynamic framework, we now turn to its implications for spacetime geometry. Jacobson famously showed that Einstein’s equations can be derived as an equation of state by applying the Clausius relation to local Rindler horizons [18]. Lemaitre interpreted the cosmological constant as a form of vacuum energy by comparing geometric terms to a perfect-fluid energy–momentum tensor [27]. This aligns with the view that spacetime geometry encodes thermodynamic constraints imposed by nested energy exchange. While Einstein’s equations relate geometry to energy–momentum, they do not specify the internal origin of $T_{\mu \nu }$. Rather than postulating fields or fluids, our framework implies, in accordance with [18], that $T_{\mu \nu }$ arises from recursively nested internal energies:

$$T_{\mu \nu }^{\mathrm{eff}}=T_{\mu \nu }\left[U^{\mathrm{loc}}(\{E_{\alpha \beta \ldots \gamma }\})\right].$$

Rather than starting from a predefined spacetime geometry, this framework suggests a reversal of the conventional approach: the energy–momentum tensor $T_{\mu \nu }$, derived from the recursive thermodynamic structure, serves as the primary object. Geometry then arises by solving the Einstein field equations with prescribed $T_{\mu \nu }$. In this view, spacetime curvature is not fundamental, but constrained by the nested energy architecture of matter.

## 6. Conclusions and Discussion

Within this thermodynamically consistent, recursive multiscale model, phenomena such as the wave function, zero-point energy, and Brownian motion arise as consequences of constrained energy exchange within nested systems. The second postulate serves as the foundational hypothesis of this framework, implying that each energy contribution on one level provides a recursively structured foundation for further decomposition. This establishes nonlinear, scale-bridging couplings that span from cosmological to quantum domains. Unlike traditional models that invoke distinct fundamental interactions at each scale, the present framework postulates a unified recursive energy structure governed by the first law of continuum thermodynamics across scale transitions. Ultimately, the wave function is reinterpreted as an effective descriptor of unresolved multiscale couplings. It no longer describes a static microstate but encodes the system’s current energetic embedding within the multiscale hierarchy. This perspective redefines locality as a scale-relative concept and opens paths to experimental access of global energetic correlations—for instance, via high-precision interferometry sensitive to cosmological background modulations; see Appendix B. Within this structure, true equilibrium states are fundamentally inaccessible, as the dynamics of the cosmic scale permanently perturb any lower-scale configuration. Thus, local equilibria can only exist transiently, stabilized by continuous energetic compensation across scales. This suggests that apparent inertial constancy may itself reflect a stabilized dynamic equilibrium, maintained by interscale energetic feedback—a hypothesis that could be tested via long-term drift analysis of precision inertial systems. Consequently, geometry itself may not be a fundamental input but a derived construct determined from the energy-momentum tensor. A full derivation of the metric remains open, but the structure suggests that the geometry is constrained by the recursive multiscale model. The model generalizes and embeds approaches such as Wetterich’s variable-mass cosmology and Verlinde’s entropic gravity into a unified energetic architecture grounded in recursive thermodynamic structure. In Wetterich’s scenario, cosmological redshift arises from increasing particle masses rather than metric expansion. This concept is extended here by permitting energy or mass modulations on one hierarchical level to induce compensatory redistributions on others, which is consistent with global conservation constraints. These interscale adjustments impose nonlocal consistency conditions, which may manifest as quantum uncertainty, relativistic inertia modulation, or effective gravitational interaction. No additional mechanisms beyond global conservation are assumed. The formalism remains fully compatible with conventional quantum theory but explores the possibility of interpreting the wave function as an effective projection of unresolved multiscale couplings.

## Figures and Tables

**Figure A1:**
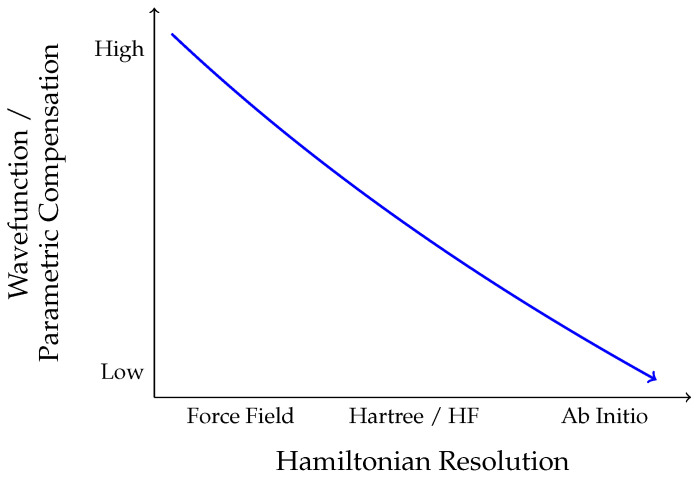
Inverse relationship between the structural resolution of the Hamiltonian and compensatory complexity in the wavefunction or parametrization.

## Data Availability

No new data were created or analyzed in this study. Data sharing is not applicable to this article.

## Appendix A. Structural Resolution and Wavefunction Compensation in Many-Body Approximations

In the theoretical framework adopted here, we systematically decompose the many-body Hamiltonian into hierarchical structural contributions. This allows us to trace which physical interactions are retained or neglected at different levels of approximation and to analyze how missing structure must be compensated elsewhere in the formalism—typically in the expressive capacity of the wavefunction or in the form of parametrized corrections.

For an *A*-electron system, we begin with the standard Hamiltonian

(A1) $$H=\sum_{\alpha =1}^{A}H_{\alpha }+\sum_{\alpha <\beta }^{A,B}P_{\alpha \beta }$$

explicitly defined as [28]

(A2) $$\begin{array}{c} E_{\alpha }=H_{\alpha }+U_{\alpha }=\frac{{\left(p_{\alpha }\nabla \right)}^{2}}{2m_{e}}-\frac{Ze^{2}}{4\pi \varepsilon_{0}r_{\alpha }}+\frac{Ze^{2}}{4\pi \varepsilon_{0}}\frac{1}{|{\vec{r}}_{\alpha }-{\vec{r}}_{\beta }|}+U_{\alpha }\;. \end{array}$$

These internal terms $U_{\alpha }$ can be recursively resolved into two- and three-body contributions:

(A3) $$U_{\alpha }=\sum_{\beta }\left(K_{\alpha \beta }+P_{\alpha \beta }+U_{\alpha \beta }\right),\;\mathrm{with}\;U_{\alpha \beta }=\sum_{\gamma }\left(H_{\alpha \beta \gamma }+U_{\alpha \beta \gamma }\right)\;.$$

The structural resolution here shows explicitly which contributions are retained or discarded in different approximation schemes. In common practice, molecular mechanics retains only the simplest local terms within $H_{\alpha }$, such as bonded interactions and fixed potentials. The remaining contributions, including $P_{\alpha \beta }$, $U_{\alpha \beta }$, and higher-order terms, are not explicitly resolved but approximated via empirical parametrizations. Hartree and Hartree–Fock methods retain $P_{\alpha \beta }$ (Coulomb repulsion), and in the case of HF, also an explicit exchange term, but they neglect $U_{\alpha \beta }$ and all three-body or higher interactions. Kohn–Sham DFT retains none of these multibody terms structurally; instead, their collective effect is embedded in a one-body exchange-correlation potential. This reveals a general structural principle:

The fewer interaction terms are explicitly resolved in the Hamiltonian, the more compensation is required by the wavefunction, effective potentials, or parametrized models.

Wavefunctions in ab initio approaches must thus develop high internal complexity to recover missing correlations. In DFT, $V_{\mathrm{xc}}^{\mathrm{ext}}$ approximates the effects of electron correlation and polarization not by explicitly resolving multi-particle operators but by embedding their averaged influence into an effective one-body potential—enabling computational efficiency at the expense of structural transparency.

Operationally, this substitution acts primarily on the αβ-scale; although the Kohn–Sham equations formally treat non-interacting particles on the α-scale, the exchange-correlation potential $V_{\mathrm{xc}}^{\mathrm{ext}}\left[\rho \right]$ encodes information originating from interactions on the αβ-level via approximate statistical averaging.

In classical force fields, the missing physics must be approximated through fixed parameters obtained from empirical or higher-level fitting. This relation is quantitatively illustrated in Figure A1.

**Remark** **A1.**
*Structural simplifications at the operator level do not remove physical effects. They are relocated within the formalism. This framework makes such redistribution transparent and allows us to identify which contributions are transferred to the wavefunction, to parametrizations, or to empirical models.*

## Appendix B. Experimental Testability

### Appendix B.1. Quantitative Drift Estimates

Our model suggests that quantum wavefunctions encode structural information about the recursively organized internal energy hierarchy of a system, which itself may undergo slow modulation through cosmological-scale thermodynamic evolution (e.g., expansion-driven energy redistribution or gravitational embedding). This opens the possibility for long-term or nonlocal experimental signatures beyond standard quantum theory. Such effects may manifest as minute, systematic drifts in spectroscopic transition frequencies, temporally evolving deviations in entanglement correlations across gravitational gradients, or spatially variant signatures in quantum state distributions prepared under nominally identical laboratory conditions. These scenarios imply a testable coupling between cosmic-scale recursive energy structures and local quantum dynamics, potentially mediated by the system’s embedding in a scale-spanning thermodynamic hierarchy.

Global conservation (Assumption 1) requires that any drift on one scale be compensated by counter-drifts on others. Recursive coupling (Assumption 2) implies that no subsystem can remain completely isolated, so local energy levels must undergo minute redistributions.

Since the only universal cosmological rate is the Hubble constant $H_{0}$, a parameterization of local drifts at the highest hierarchical level within this top-down framework results phenomenologically via the minimal, dimensionally consistent rate law

(A4) $$\frac{{\dot{U}}_{\alpha }}{U_{\alpha }}=\kappa_{\alpha }H_{0}\;,$$

with the consistency condition

(A5) $$\sum_{\alpha }\kappa_{\alpha }U_{\alpha }=0\;.$$

On lower levels, the influence is not fixed directly by $H_{0}$ but inherited recursively from the embedding subsystem. Let $i_{0}\equiv \alpha$ denote the parent level and $i_{0}\;\to i_{1}\;\to \ldots \;\to i_{L}$ an embedding chain, and let $\ell_{i}$ be a characteristic linear scale of subsystem *i*. A purely geometric, parameter-free attenuation bound then reads as

(A6) $$|\frac{{\dot{U}}_{i_{r}}}{U_{i_{r}}}|\;\leq \;\frac{\ell_{i_{r}}}{\ell_{i_{r-1}}}\;|\frac{{\dot{U}}_{i_{r-1}}}{U_{i_{r-1}}}|\;\left(r=1,\ldots ,L\right),$$

which iterates to

(A7) $$|\frac{{\dot{U}}_{i_{L}}}{U_{i_{L}}}|\;\leq \;\frac{\ell_{i_{L}}}{\ell_{i_{0}}}\;|\frac{{\dot{U}}_{i_{0}}}{U_{i_{0}}}|\;=\;\frac{\ell_{i_{L}}}{\ell_{\alpha }}\;\left|\kappa_{\alpha }\right|\;H_{0}.$$

No additional universal parameter is required; the observable rate on microscopic scales is a small, modulated fraction of the top-level rate that naturally decreases with depth. Thus, the observable rate on microscopic scales is a small, modulated fraction of the top-level rate that decreases with depth, which is consistent with the non-observation of large drifts in bound systems such as the Earth, leaving only tiny differential signatures accessible to precision spectroscopy. The coefficients $\kappa_{\alpha }$ encode the effective embedding of a given subsystem into the global redistribution and are constrained by the consistency condition. In this sense, they represent structural response factors rather than free variables, and their values are subject to direct experimental determination through precision spectroscopy. While standard drift hypotheses envisage a monotonic change, the present framework predicts compensatory, cyclic redistributions—the “cosmic hum” of recursive energy exchange across scales rather than a uniform secular trend. It should be emphasized that the linear form Equation (A4) characterizes only the effective local drift of a single subsystem. When considering the full recursive coupling, the global dynamics are inherently nonlinear, since the compensatory condition constrained by Equation (A5) couples all subsystems and enforces nontrivial redistributions across scales. Thus, linearity is a local approximation, while the global picture is nonlinear by construction. In contrast to phenomenological drift hypotheses, e.g., variable-mass cosmologies, the present framework does not assume variability but derives it as a structural consequence of global conservation and recursive embedding; moreover, monotonic variation is excluded by ${\dot{U}}_{0}=0$, enforcing compensatory patterns across scales. It should be stressed that no universal drift applies equally to all subsystems, since such a drift would be unobservable. Instead, only relative shifts matter, governed by differences in the effective coefficients $\kappa_{\alpha }$. While $H_{0}$ provides the universal reference scale, the compensatory condition given by Equation (A5) ensures that the net drift averaged over all subsystems vanishes, so global conservation is preserved. Observable signatures, therefore, always appear as relative drifts of order $H_{0}$, rather than as a uniform monotonic change. Hence, the framework is not excluded by present limits but quantitatively constrained, with ongoing advances in precision spectroscopy expected to further refine the allowed parameter space for $\kappa_{\alpha }$.

For a transition $\nu_{nm}=\left(E_{n}-E_{m}\right)/h$ at microscopic scale $\ell_{\mathrm{micro}}$, one has

(A8) $$|\frac{{\dot{\nu }}_{nm}}{\nu_{nm}}|\;≲\;\frac{\ell_{\mathrm{micro}}}{\ell_{\alpha }}\;\left|\kappa_{\alpha }\right|\;H_{0},$$

(up to an order-unity geometric factor for non-near-degenerate levels).

This provides the direct bridge from the hierarchical bound to the spectroscopic observable used below.

Taking the host galaxy’s virial size as the top scale, $R_{\mathrm{vir}}\;\approx \;200\;\mathrm{kpc}\approx 6.17\times 10^{21}\;m$, and microscopic lengths $a_{0}=5.29\times 10^{-11}\;m$ (atomic), $\lambda_{C}\left(e\right)=2.43\times 10^{-12}\;m$ (electronic), and $1\;\mathrm{fm}=10^{-15}\;m$ (nuclear), the geometric ratios are

(A9) $$\begin{array}{c} \frac{\ell_{\mathrm{micro}}}{\ell_{\alpha }}=\left\{\;8.6\times 10^{-33},\;3.9\times 10^{-34},\;1.6\times 10^{-37}\;\right\}\;. \end{array}$$

Inserting this into Equation (A8) with $H_{0}≃7.0\times 10^{-11}\;{\mathrm{yr}}^{-1}$ and $C_{nm}=O\left(1\right)$ (non-near-degenerate) yields the conservative ceilings

(A10) $$\begin{array}{c} |\frac{\dot{\nu }}{\nu }|\;≲\;\left\{\;6.0\times 10^{-43},\;2.8\times 10^{-44},\;1.1\times 10^{-47}\;\right\}\;\left|\kappa_{\alpha }\right|\;{\mathrm{yr}}^{-1}\;. \end{array}$$

For a representative optical transition ($\nu_{0}\;\approx \;4.29\times 10^{14}\;$ Hz, Sr), this implies

(A11) $$\begin{array}{c} |\dot{\nu }|\;≲\;\left\{\;2.6\times 10^{-28},\;1.2\times 10^{-29},\;4.9\times 10^{-33}\;\right\}\;\left|\kappa_{\alpha }\right|\;\mathrm{Hz}/\mathrm{yr}\;. \end{array}$$

For completeness, a naive $H_{0}$-level comparison without geometric attenuation gives $|\kappa_{\alpha }|≲1.4\times 10^{-8}$ from $|\dot{\nu }/\nu |<10^{-18}\;{\mathrm{yr}}^{-1}$, whereas the microscopic, parameter-free statement is the length-scale ceiling above.

These values show that purely geometric attenuation suppresses microscopic drifts far below current clock sensitivities, which is consistent with the non-observation of large effects in bound systems.

Thus, even under maximal host-level response, purely geometric attenuation bounds microscopic drifts far below current sensitivities; any realistic recursive coupling reduces them further.

For completeness, we note that a naive $H_{0}$-based comparison—taking $\dot{\nu }/\nu ∼H_{0}$ without geometric attenuation—implies from current clock limits $|\dot{\nu }/\nu |<10^{-18}\;{\mathrm{yr}}^{-1}$ a separate top-level bound $|\kappa_{\alpha }|≲1.4\times 10^{-8}$, whereas the precise microscopic statement is the parameter-free length-scale ceiling derived above.

### Appendix B.2. Geochemical Barcoding

Furthermore, geochemical barcoding of volcanic samples from the Afar triple junction reveals temporally periodic mantle upwelling pulses with characteristic recurrence intervals of 50–150 kyr [29]. These pulses, preserved in isotopic and trace-element stratigraphy, provide direct evidence for rhythmic deep-Earth convection. Their consistent frequency ($10^{-5}$ Hz) and variable expression across rift zones suggest modulation by lithospheric structure and spreading dynamics. Within the proposed model, such pulsations may reflect macroscopic cyclicities in planetary energy distribution, which may modulate deep Earth convection via long-wavelength strain fields or energy density fluctuations consistent with the proposed recursive multiscale coupling framework. The Afar system thus offers a rare empirical proxy for testing large-scale oscillatory behavior in geodynamic and possibly cosmological contexts.

### Appendix B.3. Brownian Motion

We propose that Brownian motion, long seen as the outcome of local stochastic collisions, can be reinterpreted within the framework of our recursive motion Equation (16) as a macroscopic projection of nonlinear multilateral interactions across nested cosmic structures. In this view, deviations from classical Langevin dynamics observable through high-resolution particle tracking are not mere stochastic noise but scale-modulated fluctuations encoding the influence of recursively coupled energy redistribution, reflecting multiscale energy coupling across cosmological embedding. Brownian trajectories thus carry structural imprints of the universe’s dynamics, much like the quantum wave function at the quantum level. By analyzing fluctuation spectra, autocorrelations, and force-response patterns in varied environments, one can extract information about the recursive energy coupling governing motion at mesoscopic scales. This approach opens a new route to probe the thermodynamic structure of the universe experimentally, quantitatively, and from within.
